# Views on virtual education during the COVID-19 pandemic among medical and paramedical students in India

**DOI:** 10.6026/97320630018518

**Published:** 2022-06-30

**Authors:** Yuvaraj Maria Francis, PK Sankaran, CP Kirthika, Balaji Karunakaran, S Sathish Kumar, D Karthikeyan, Madhan Krishnan, Shyamaladevi Babu

**Affiliations:** 1 Department of Anatomy, Saveetha Medical College, Saveetha Institute of Medical and Technical Sciences, Thandalam, Chennai- 602 105, India; 2 Department of Anatomy, All India Institute of Medical Sciences, Mangalagiri -52008, Andhra Pradesh, India; 3 Department of Anatomy, Sri Ramachandra Institute of Higher Education and Research, Porur, Chennai : 6000116, India; 4 Department of Community Medicine, Vinayaka Missions Medical College and Hospital, Karaikal, Pondicherry, India; 5 Department of Microbiology, Vinayaka Missions Medical College and Hospital, Deemed university, Karaikal, Pondicherry, India; 6 Faculty of Allied Health Sciences, Chettinad Hospital and Research Institute, Chettinad Academy of Research and Education, chengalpattu- 603103 India

**Keywords:** COVID 19 pandemic, Education, Medical students, Perceptions, Virtual learning and teaching

## Abstract

The COVID-19 pandemic has made the educational institutions to implement the mandatory virtual learning in medical education. It is undeniable that electronic gadget aided learning have a significant role to play during a pandemic. Both faculty and students are getting accustomed to this 'New Normalcy'. Therefore, it is of interest to determine the effectiveness and perception of virtual teaching and learning during the COVID 19 pandemic. A cross-sectional study was conducted in which 336 medical and 336 paramedical students of both the genders with age group of 17 - 21 years participated. The data obtained were analyzed using the SPSS software. The shift from class room teaching to virtual learning has led to many health issues among students such as eye strain, anxiety, depression, musculoskeletal problems and obesity. The students also had inadequate time to interact with faculties. Data shows that virtual learning an alternative mode to traditional method during a pandemic.

## Background:

The World Health Organization (WHO) declared COVID-19 as pandemic and it was considered a public health emergency of international concern. The lockdown imposed following it affected the education system across the world and brought profound organizational changes in the traditional method of teaching [1]. Covid-19 crisis created a stress among all the individuals from younger to older generation especially the teaching faculties and students. The closure of institutions stopped their conventional teaching method in order to maintain social distancing [2]. Both WHO and UNESCO observed 92% interruption in learning across the world with ceasing of midday meal in developing countries like India. Many of the educational institutions in India shifted from conventional to virtual teaching which became a challenge for both faculties and students, particularly in health education due to lack of hands-on trainings [3, 4]. The virtual teaching and learning has a wide set of applications and processes, through electronic tools to deliver education and training. The benefits are it is suitable for everyone, they can learn at the ease of their flexible timing, and online content such as videos and lectures can be seen multiple number of times and students can learn at their own pace. The drawbacks are increase in the screen timing which in turn lead to adverse effects on vision, the neck and back ache due to improper posture [5,6]. Therefore, it is of interest to document views on virtual education during the COVID-19 pandemic among medical and paramedical students in India.

## Methods:

### Ethical clearance:

This cross-sectional survey was done after obtaining Institutional Ethical clearance from Vinayaka Missions Medical College & Hospitals, Karaikal (VMMC/MICRO/2021/82).

### Dataset:

The participants were 672 students (336each from medical and paramedical) from Vinayaka Missions, Saveetha Medical College. Among them, 453 were females and 219 were males. The responses obtained in the study were little higher, to reduce the standard error we included same number of responses. A self-designed and validated questionnaire was prepared in Google form, which fulfilled the criteria of the research and it was analyzed by peer faculties, before sharing with the participants. The questionnaire was categorized in five subdivisions such as

1. Informed Consent,

2. Socio-demographic details,

3. Questions about perception and attitude of student towards virtual teaching learning,

4. Questions about methods used for virtual teaching learning including gadgets and online platform used,

5. Questions about health problems arising due to virtual learning.

The Google form was distributed to the students through the WhatsApp, Telegram and the personal e-mail addresses. The students voluntarily participated in the study and their confidentiality was maintained.

### Statistical analysis:

The data were collected, organized and analyzed statistically through chi-square test and logistic regression analysis through SPSS software, version 21.P value of less than 0.05 was taken as significant.

## Results:

### Socio-demographic data:

On the whole, 672 participants responded (excluding 9 duplicates) with the response rate (672/930=72%). About 336participants each from medical and paramedical actively participated in this study. Among them, 453 (67.4%) were females and 219 (32.6%) were males with a median age of 18 (17 - 21 year old). The majority of the participants were residence from urban area 440 (65.5%)who had easy availability of Internet facility. The basic facilities required for attending the virtual learning such as web provision, gadgets, efficiency in handling gadgets and usage of internet, determines the feasibility of virtual learning. The vast majority of the participants use smartphone 609 (90.6%) for their virtual classes, while rest of them use laptop. About 97.8% of the individuals have sound knowledge in handling the gadget. More than half of the participants (51.93%) possess WIFI for their online classes where 98.5% of them have good knowledge in using internet source. The online platforms used for E-learning were Cisco webex (77.9%), Google meet (16.96%), ZOOM meeting (5.05%), Microsoft Team and GoTo Meeting are among others. All these parameters were dependent on the affluence of the family and the geographical location of the students (Table, 1 - see PDF). There were also significant differences between medical and paramedical students concerning these parameters.

### Virtual learning and factors influencing:

Teaching methods employed in E-learning: There are number of teaching methods available to train and educate the students, to improve their knowledge and creativity. The gold standard and commonly used teaching method for demonstration by faculties is power point presentation (89.4%) among the medical (47.9%), and paramedical students (41.5%) (Figure 1 - see PDF). The remaining teaching methods used in the virtual learning were monotonous lectures without power point presentation (28.4%), video-based teaching (73.2%), recorded lectures (39.3%), quizzes (50.0%), virtual models in classes (39.4%), online whiteboard teaching with diagrams (31.5%). and interactive sessions (67.6%). The interactive sessions facilitated the medical students to interact better than the paramedical students. The medical and paramedical students preferred 3-6 classes/day with duration of each class being not more than 45 minutes, with 15 minutes gap between the sessions, and also they were in need of interactive sessions after the classes for 15 minutes to clarify the doubts. About 97.5% students preferably asked their faculties to share their study materials and informed them to conduct quizzes, jigsaws and virtual models.

### Duration of classes:

The students both from medical and paramedical courses had regular virtual theory sessions, and 48% students had virtual practical sessions and 73.3% had virtual internal assessments. The session and duration of classes were significantly higher among medical students in comparison to paramedical students. Breaks between sessions were infrequent in both medical and paramedical students, wherein 15% of the students reported that they had no breaks between sessions. The students both medical and paramedical did not have sufficient time to interact with the faculties during virtual learning to clarify their doubts.

### Health issues faced by students during online classes:

The students have faced various kinds of health issues such as eye strain (58.60%) which was frequently experienced, followed by back pain (55.70%), neck pain (55.40%), head ache (54.20%), sleep disturbances (43.80%), obesity (47.5%) and fatigue (39.30%) (Figure 2 - see PDF). In addition to that, students also expressed that concentration was affected to a great extent in virtual learning than traditional class room teaching. The medical students expressed significantly more health issues comparatively with the paramedical students owing to increased screen time. The expanded period of online classes in a day and longer period of each sessions resulted in a higher proportion of students to develop various health issues with circadian rhythm disturbances.

Perceptions and attitudes of medical and paramedical students towards e-learning: The perceptions and attitudes differ between medical and paramedical students. The majority of the students (87.5%) felt that the COVID 19 pandemic has affected the education, health and circadian rhythm, among them medical students were 82% and paramedical students were 75% (Figure 2 - see PDF). Overall, only 37.4% of the students felt that virtual learning is a best alternative to conventional class room teaching to continue education during the COVID 19 pandemic situations, but students felt that life style modification leads to changes in the health and biological clock. The students undergoing posting in clinical department had faced lot of difficulties such as observation, examination of patient etc, than the first year students. The students who had access to separate internet resource and good efficiency in handling were more likely to cope with the e-learning in comparison with traditional face to face teaching. The students from urban area had a separate room and adequate facility for attending virtual learning classes without much hindrance, in contrast to this, the students from rural area faced many difficulties in attending the online sessions and the reasons for hindrances being lack of separate room, web resources, gadgets and power fluctuation. The students also benefited by attending webinars that were conducted by countries across the globe, which helped them to gain knowledge and improve their skills. Nevertheless, e-learning methods had no scope for extracurricular and co-curricular activities for the students, which led to stress and obesity among them, which can further lead to various kinds of diseases in the future.

## Discussion:

The COVID-19 pandemic has affected around 216 countries involving 5.5 million people. This has led to closure of educational institutions causing disturbances in student's education. Based on the need to maintain continuity in imparting knowledge to students, the educational institutions initiated virtual learning for students. The SWAYAM (Study Webs of Active-Learning for Young Aspiring Minds) an integrated web portal, conducts virtual education for university students. However, being a developing country, rural areas of India, still lacks reliable network and web resources. Most of the students from rural area cannot utilize online learning to its full potential. This survey based research shows that students from urban region preferably showed better experience in virtual learning, through facilities such as good web resources, effective online platforms, availability of electronic gadgets, and proficiency in handling gadgets or web resources. Power-point presentation was the most frequently used method for demonstration and teaching. Students experienced that they had inadequate time to interact with faculties during the virtual sessions and they faced many difficulties in virtual sessions such as lack of interaction and physical examinations with patients, lack of gadgets etc. It was observed that students who attended virtual classes for more than 5 hours a day, with a lack of extracurricular activities faced lot of health problems.The COVID-19 crisis has showed the remarkable significance and need for digital devices, technology and internet resources among the developing countries like India and developed countries. The internet resources and gadgets play a vital role in many fields especially in teaching. About 72% of India’s population lives in rural areas and they face various kinds of hindrances. However, only 15.7% have access to the web resources in rural areas compared to 45% in urban areas because service providers are reluctant to invest in small towns and villages [7]. This study also observed that the students from rural areas faced many problems such as lack of gadgets, inefficiency in handling gadgets and online platforms, power fluctuations, and non-availability of web resources for the virtual learning purpose during the COVID-19 crisis. Similar to our research, Baczek et al also observed the problems faced by medical students during e-learning among Polish population [8].

Learning is an intellectual process of transforming information and experience into a relatively permanent change in ones skills, knowledge, behaviour and attitude. The three important skills of learning are cognition, psychomotor and affective. Implementation of Bloom’s taxonomy in virtual learning will help the faculties to construct and create ideas to make their classes more effective [9]. The orientation and understanding of the three domains of learning will differ among the students. These domains of learning will be efficiently delivered to the students while undergoing face-face learning but the faculties face difficulty while delivering the same through virtual session. The methods employed in virtual teaching include instructional videos, case-based learning, quizzes, small group learning, online skill simulations, concept mapping, jigsaw, role play, researches, seminars, webinars, webcasts, internal assessments and power point presentation [10]. Similarly, in this study we have also included various innovative methods for virtual learning, to maintain the continuity in learning for students. Also, this kind of sudden shift from face- face teaching to virtual mode of teaching in educational system had not been faced earlier, by faculties and students. The initiation of this kind of virtual learning can be beneficial only under the pandemic situations like COVID -19. The medical and paramedical faculties have a dual role in their profession like teaching students and also taking care of patients, especially in emergency cases, due to which faculties face lack of time to prepare and organize for their virtual learning sessions. In a study conducted by [11], reveals that, 50% of the students have showed that the three domains of learning and teaching are fulfilled in traditional classroom teaching in comparison with virtual teaching. Similarly, this study showed that 87.3% students were satisfied only with face-face traditional teaching and the remaining 13.7% of students agreed that virtual learning can be used only as a substitute in the pandemic situation. Only 67.7% of the students acknowledged that they have adequate time to interact with the faculties. Also, 15% of the students felt that they did not have adequate gap between the virtual sessions. The students also faced frequent problems while undergoing virtual learning such as internet disconnection, telephone calls, power fluctuation, battery drainage which affected the three domains of learning. In concurrence with above problems students from west Bengal has also faced similar problems while undergoing e-learning session during the COVID 19 pandemic [12]. The commonly used applications are ZOOM, Microsoft Teams, cisco-Webex, Google Classroom and other platforms are also used by various institutes for teaching and research oriented purposes. Each platform has unique features and most of them are available free of cost for basic use [13]. In the present study, Cisco webex platform was frequently used among various institutes, followed by zoom and Google meet. Cisco-webex has high end connectivity, ease of use, good security, interactivity, and features such as screen sharing, video recording option [14]. The concentration and orientation of the learners mainly depends on encouragement, duration of session, crystal clear explanation of topics which make it easy to learn and understand. This kind of teaching helps students to improve knowledge, creativity, research, and career. The students who complete schooling and enter a new profession such as medical and paramedical will come across new terminologies and need adequate time for grasping and understanding. While learning new subjects they acquire new information from every lecture and also need adequate break between sessions, for information to be processed with the help of higher intellectual centers [15,16]. The understanding and orientation of session can be achieved only through, short duration of sessions, small group teaching, video presentation, model making, jigsaw, concept mapping, role play, and quizzes [17].

Around 60-70% percentage of the learners faced health problems due to extended screen time in e-learning sessions. It has been observed that excessive usage of gadgets, and abnormal posture can affect the students both physically and psychologically [18,19]. In this study, learners have reported various health problems such as 58.60 % had eyestrain, 55.70% showed posture related problems, 43.80% showed circadian changes and few students showed lack of concentration and orientation and increase in body weight. The health issues faced by students are mainly due to factors such as, on screen time, loss of activity and improper ergonomics. The eyestrain could be due to increased use of gadgets continuously and changes in biological clock [20]. The usage of gadgets in various postures and reduced locomotion could be the major reasons for developing musculoskeletal issues such neck pain, low backache, headache, and shoulder or wrist pain. The biological clock plays a vital role in humans, to stay healthy and maintain a peaceful life [21]. Melatonin is a hormone secreted by the pineal gland which helps to maintain the circadian rhythm [22]. The usage of digital devices emits blue light from the diodes and alters the biological cycle and results in eyestrain [23]. Frequent use of digital devices and web resources can debilitate development of higher intellectual functions in students, especially concentration and orientation which play a vital role in learning. Mulugeta et al, showed that there is increased in body weight among students especially in females in comparison males during COVID 19 pandemic lockdown is due to lack of activities [24].There are many studies done, based on the perceptions of students towards e-learning during the COVID 19 pandemic, but there are no comparative studies between medical and paramedical students in South Indian region. The limitations of this study are collection of data only by online method; hence significantly fewer participants took the survey.

## Conclusion:

High internet charges, inadequate internet connectivity, a lack of technical abilities in using e-learning platforms, and difficulties in obtaining energy are typical impediments to students' access to e-learning. It is also advised that medical and paramedical students, as well as teachers, be educated and trained on how to use existing e-learning platforms. The study concludes that virtual learning is an alternative mode to traditional face – face teaching in pandemic situations.
